# The structural and photosynthetic characteristics of the exposed peduncle of wheat (*Triticum aestivum *L.): an important photosynthate source for grain-filling

**DOI:** 10.1186/1471-2229-10-141

**Published:** 2010-07-11

**Authors:** Lingan Kong, Fahong Wang, Bo Feng, Shengdong Li, Jisheng Si, Bin Zhang

**Affiliations:** 1 Crop Research Institute, Shandong Academy of Agricultural Sciences, 28 Sangyuan Road, Jinan 250100, Shandong, China

## Abstract

**Background:**

In wheat (*Triticum aestivum *L), the flag leaf has been thought of as the main source of assimilates for grain growth, whereas the peduncle has commonly been thought of as a transporting organ. The photosynthetic characteristics of the exposed peduncle have therefore been neglected. In this study, we investigated the anatomical traits of the exposed peduncle during wheat grain ontogenesis, and we compared the exposed peduncle to the flag leaf with respect to chloroplast ultrastructure, photosystem II (PSII) quantum yield, and phospho*enol*pyruvate carboxylase (PEPCase; EC 4.1.1.31) activity.

**Results:**

Transmission electron microscope observations showed well-developed chloroplasts with numerous granum stacks at grain-filling stages 1, 2 and 3 in both the flag leaf and the exposed peduncle. In the exposed peduncle, the membranes constituting the thylakoids were very distinct and plentiful, but in the flag leaf, there was a sharp breakdown at stage 4 and complete disintegration of the thylakoid membranes at stage 5. PSII quantum yield assays revealed that the photosynthetic efficiency remained constant at stages 1, 2 and 3 and then declined in both organs. However, the decline occurred more dramatically in the flag leaf than in the exposed peduncle. An enzyme assay showed that at stages 1 and 2 the PEPCase activity was lower in the exposed peduncle than in the flag leaf; but at stages 3, 4 and 5 the value was higher in the exposed peduncle, with a particularly significant difference observed at stage 5. Subjecting the exposed part of the peduncle to darkness following anthesis reduced the rate of grain growth.

**Conclusion:**

Our results suggest that the exposed peduncle is a photosynthetically active organ that produces photosynthates and thereby makes a crucial contribution to grain growth, particularly during the late stages of grain-filling.

## Background

Green leaves have commonly been considered the primary source of photosynthate production. In addition to their leaves, higher plants can potentially use almost any vegetative or reproductive structure to perform photosynthetic CO_2 _assimilation for growth and development [1-3]. In wheat, there are a number of non-foliar organs that are photosynthetically active, including all parts of the ear and the exposed part of the peduncle, all of which can assimilate CO_2 _when they are exposed to light [4,5]. In an investigation into the effects of defoliation of spring wheat on grain yield, Rosyara et al. (2005) found that the yield reduction was lower than expected when the upper two leaves were removed for all genotypes examined [6]. They proposed that the photosynthetic behaviour of other sources played a compensatory role in stabilising the yield. An accumulating body of evidence suggests that non-foliar green organs may contribute as much as 40-50% of the photosynthates required by developing wheat grains [5,7,8].

The peduncle, which is located at the first internode directly below the spike, has a diversity of critical roles in crop productivity. Development of the vascular system in the peduncle is essential for transporting assimilates to the filling grain [9]. Elongation of the exposed part of the peduncle lessens the risk of leaf-borne pathogen infections in the ear by increasing the distance between the upper leaves and the ear [10]. Under drought stress or high temperatures, this organ (and in particular the exposed part) maintains significantly higher water potential than the flag leaf [11]. The upper part of the peduncle develops leaf-like autotrophic carbohydrate metabolism when it is exposed to high irradiance [12], accounting for a high proportion of the photosynthesis of the stem [5,7]. Wang et al. (2001) suggested that photosynthesis in the exposed peduncle and flag leaf sheath contribute about 9-12% of grain dry mass, depending on the wheat cultivar [5]. Although the exposed peduncle has been identified as one of the photosynthetically active organs in wheat, the structure of the chloroplasts and the functional events related to photosynthesis and the translocation of photosynthates during the period of grain-filling have not precisely characterised. Evidence from ultrastructural observations and photochemical assays is needed to elucidate the mechanisms by which the exposed peduncle produces photoassimilates for grain development.

The thylakoid membranes in chloroplasts accommodate all of the molecular complexes that perform the light-driven reactions of photosynthesis and provide a medium for energy transduction. PSII is one of the key complexes clustered in the appressed regions of the granum stacks in the thylakoid membrane system [13,14]. The integrity and organisation of the thylakoid membranes in chloroplasts are essential for photosynthesis. Little information is available on the chloroplast ultrastructure of exposed peduncles or their temporal variations in photochemical efficiency during grain-filling. PEPCase functions as a primary carboxylase in the cytoplasm, fixing ambient CO_2_, re-fixing respiratorily released CO_2 _and then producing the carbon skeleton for the tricarboxylic acid cycle [3,15-17]. Monitoring the temporal changes in PEPCase activity is therefore crucial for evaluating photosynthetic assimilatory capacity in plants.

The purpose of this study was to investigate the photosynthetic features of the exposed part of the wheat peduncle and its photosynthetic contribution to grain-filling. The flag leaf was used as a reference since it is the main source of photosynthate for the developing grain [18,19]. Particular attention has been given to the anatomical, ultrastructural and photo-physiological characteristics of the exposed peduncle that contribute to its photosynthetic performance.

## Results

### Growth and anatomy of the exposed peduncle

At stage 1, the peduncle length was 19.6 ± 2.3 cm (n = 30), and 7.8 ± 0.8 cm (n = 30) of this length was exposed above the flag leaf sheath. The peduncle continued to grow at a mean rate of about 14 mm d^-1 ^until the eighth day after anthesis. At this time, the peduncle reached a final length of 30.6 ± 4.1 cm (n = 30), of which 12.4 ± 2.2 cm (n = 30) was exposed. At maturity, the diameter of the exposed peduncle was 3.14 ± 0.32 mm (n = 30) at the basal end and 2.72 ± 0.25 mm (n = 30) at the upper end (base of the panicle).

**Figure 1:**
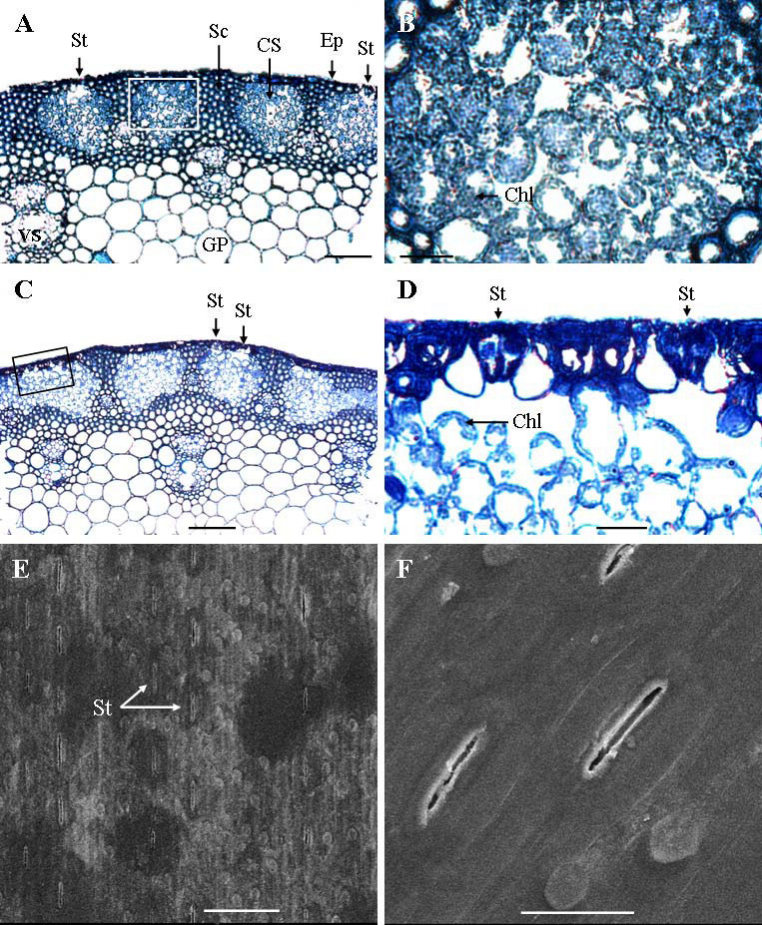
**Anatomical features of the wheat exposed peduncle**. (A) Semithin section showing the anatomical structure of the centre of an exposed peduncle at stage 2 (the milk-development stage). (B) Higher magnification of the area highlighted in (A) showing chlorenchyma cells that are rich in chloroplasts. (C) Semithin section showing anatomical structure of the middle part of an exposed peduncle at stage 4 (the hard dough-development stage). (D) Magnified micrograph of the pane in (C) showing the decline in the number of chloroplasts in the chlorenchyma cells and stomata on the epidermis. (E) Scanning electron microscope (SEM) images of the middle part of an exposed peduncle showing that the stomata are arranged axially in rows. (F) SEM images showing the morphology of the stomata. Chl: chloroplast, CS: chlorenchyma strand, Ep: epidermis, GP = ground parenchyma, Sc: sclerenchyma, St: stomata, and VS = vascular strand. Bars: (A and C), 200 μm; (E), 100 μm; (B, D and F), 30 μm.

Cross-sections of the exposed wheat peduncles showed the epidermis, sclerenchyma, chlorenchyma strands, ground parenchyma, and the vascular strands (Figure 1A and 1C). The chlorenchyma strands were composed of cells rich in chloroplasts at the early stages (Figure 1B). The number of chloroplasts in the chlorenchyma cells was reduced at stage 4 (Figure 1D). Stomata occurred frequently (Figure 1A and 1C) and in rows that were arranged longitudinally along the stem (Figure 1E and 1F). The average stomatal density reached 80.23 ± 9.41 mm^-2 ^on the exposed part of the peduncle (Figure 1E and 1F). This measure was significantly higher than the density measured on the adaxial face of the flag leaf (58.14 ± 7.25 mm^-2^) and the density recorded on the abaxial face of the flag leaf (45.65 ± 6.43 mm^-2^).

### Chloroplast structure related to grain development

**Figure 2:**
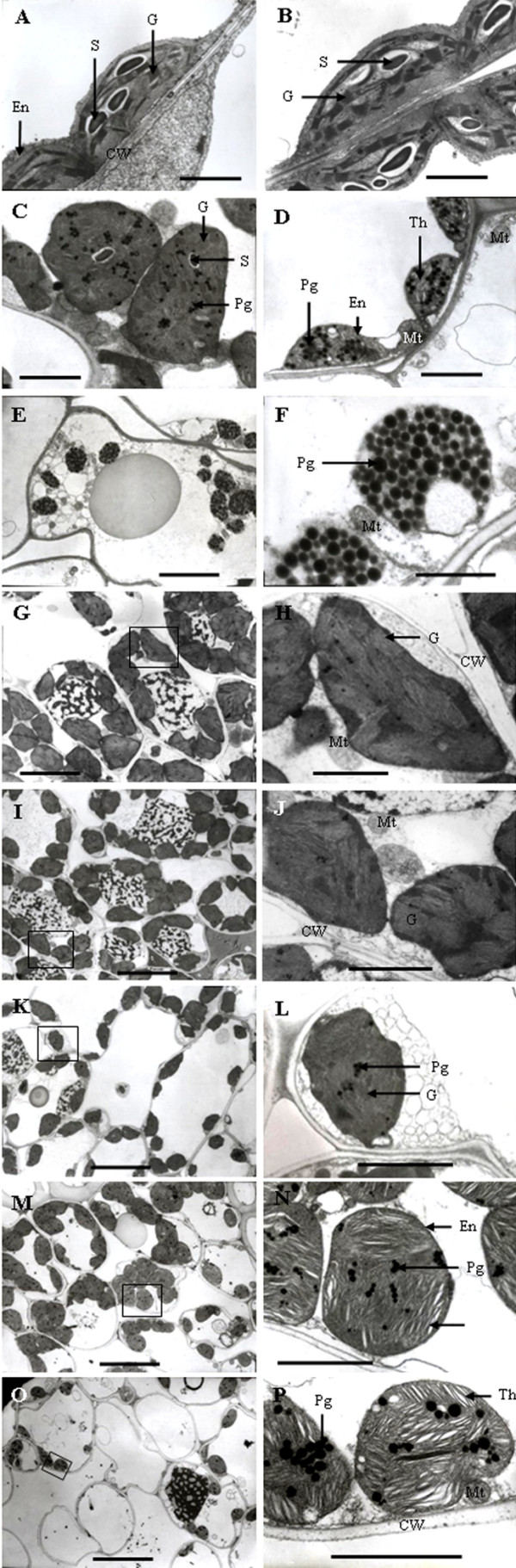
**Transmission electron micrographs showing the ultrastructure of the chloroplast at different stages in flag leaves (A-F) and exposed peduncles (G-P)**. (A, G and H): stage 1; (B, I and J): stage 2; (C, K and L): stage 3, (D, M and N): stage 4; (E, F, O and P): stage 5. (H), (J), (L), (N) and (P): higher magnification of the area highlighted in (G), (I), (K), (M) and (O), respectively. Stages 1-5 correspond (respectively) to the end of anthesis, the milk-development stage, the soft dough-development stage, the hard dough-development stage and the ripening stage. Bars: (A-D), 2 μm; (E, G, I, K, M, O), 5 μm; (F, H, J, L, N, P), 1 μm. CW, cell wall; En, envelop; G, granum; Mt, mitochondrion; Pg, plastoglobuli; S, starch; Th, thylakoid.

Figure 2 shows transmission electron micrographs of chloroplasts in flag leaves and in exposed peduncles; each is typically representative of the ultrastructural regime of more than 80% of the chloroplasts in four plants. At stages 1 and 2, the cells of the flag leaves had well-differentiated chloroplasts, containing fully developed grana with numerous layers and well-developed stroma lamellae with several starch granules (Figures 2A and 2B). At stage 3, the shape of the chloroplasts changed from lens-like to ellipsoid or round. Although the number of grana did not seem reduced in most chloroplasts, the thylakoid membranes began to dilate slightly; this process was accompanied by an irregular arrangement of the thylakoid stacks, an apparently declining amount of starch, and a marked increase in the number of plastoglobuli (Table 1). All of these changes indicate the onset of total chloroplast degradation and leaf senescence (Figure 2C). At stage 4, the chloroplasts were smaller but structurally swollen, and their ultrastructure was characterised by a disintegrating envelope, disrupted and irregularly-shaped thylakoids and irregularly-arranged thylakoid grana with fewer stacks. A concurrent decline in the number of chloroplasts was also evident (Figure 2D; Table 1). At stage 5, the chloroplasts were characterised by completely disintegrated thylakoid membranes and a considerable accumulation of plastoglobuli of approximately the same size. The entire structure of the chloroplasts was ruptured (Figure 2E and 2F; Table 1).

**Table 1:** Temporal changes in the chloroplast ultrastructure of the flag leaf and the exposed peduncle of wheat.

Developmental stage^1^	Number of chloroplasts per mm^2 ^cell profile		Number of starch grains per chloroplast profile		Number of plastoglobuli per chloroplast profile	
	
	Flag leaf	Exposed peduncle	Flag leaf	Exposed peduncle	Flag leaf	Exposed peduncle
1	68.02 ± 7.16a^2,5^	89.95 ± 8.12a	2.21 ± 0.38a^3^	0	7.83 ± 2.34d^4^	9.82 ± 2.08c
2	65.47 ± 6.71a	87.30 ± 8.54a	2.27 ± 0.24a	0	10.69 ± 2.79d	12.27 ± 3.03bc
3	56.85 ± 5.44b	76.95 ± 6.83b	1.24 ± 0.31b	0	24.74 ± 5.64c	14.60 ± 4.14b
4	33.86 ± 4.29c	55.84 ± 6.47c	0.28 ± 0.10c	0	42.56 ± 7.43b	19.65 ± 4.37a
5	14.42 ± 2.02d	21.28 ± 2.59d	0 ± 0d	0	70.44 ± 11.83a	23.49 ± 3.56a

^1 ^Stages 1-5 correspond to the end of anthesis, the milk-development stage, the soft dough-development stage, the hard dough-development stage and the ripening stage, respectively.

^2 ^Values given are the mean ± standard deviation for four plants (>10 mm^2 ^of sections were recorded from each plant).

^3 ^Values given are the mean ± standard deviation for four plants (>30 cells with the largest dimension were recorded from each plant).

^4 ^Values given are the mean ± standard deviation for four plants (>50 chloroplasts with the largest dimension were recorded from each plant).

^5 ^Means followed by different letters within a column are significantly different by a Tukey test (*P *< 0.05).

In the exposed peduncles, the cells contained well-differentiated chloroplasts at a higher density than those of the flag leaves at stage 1. Although the chloroplasts were not regular in shape and the granum arrangement was not regular, the chloroplasts were full of a large number of thylakoids and the grana lamellae were well-organised and clearly discernible. Unlike the flag leaves, the exposed peduncles lacked starch granules in the chloroplasts (Figure 2G and 2H; Table 1). At stage 2, no notable changes were observed in the ultrastructure or the shape of the chloroplasts (Figure 2I and 2J). At stage 3, even though the number of chloroplasts decreased and nuclei in some cells disappeared, the chloroplasts contained a large number of thylakoids and the matrix of the chloroplasts was still dense. At this stage, the number of plastoglobuli increased noticeably and numerous small vesicles or membrane-like fragments were observed in the cytoplasm (Figures 2K and 2L). At stage 4, the chloroplasts became spherical, and an apparent increase in the plastoglobulus content was observed. Although the nucleus disappeared in almost all of the cells, the membranes constituting the thylakoids were still very distinct and plentiful. The most striking changes occurred in the structure of the thylakoids, i.e., loss of the parallel arrangement of the grana lamellae became evident in some chloroplasts, and some of the thylakoids became swollen (Figure 2M and 2N). At stage 5, the number of chloroplasts decreased noticeably (Table 1); the envelope and the thylakoids of the grana were indistinctly discriminated one from another. However, the grana lamellae were still abundant in comparison to the complete disruption of the thylakoid membrane system in the flag leaves (Figure 2O and 2P).

### Chlorophyll fluorescence

**Figure 3:**
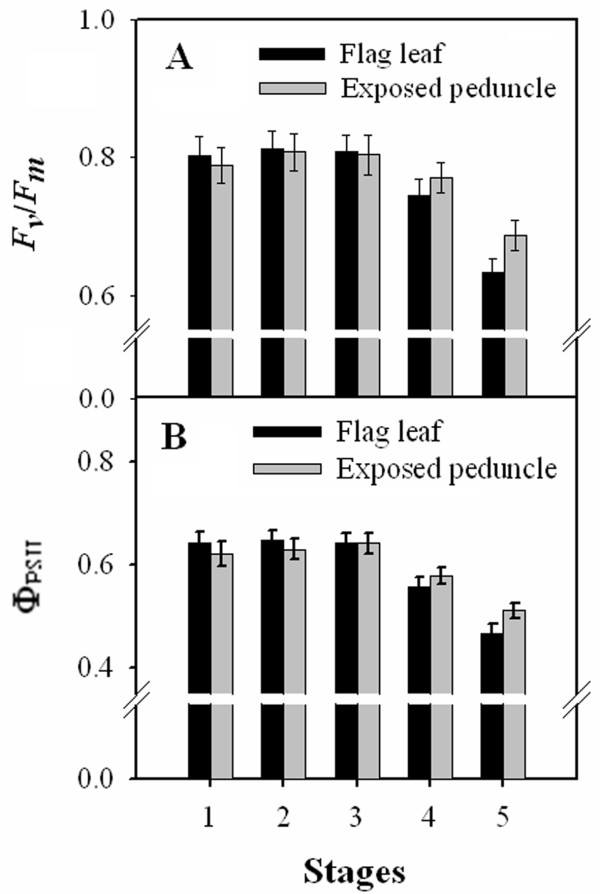
**Changes in the chlorophyll fluorescence parameters *F_v_/F_m _*(A) and Φ_PSII _(B) in flag leaves and exposed peduncles at different stages**. Each data point in the figure represents the average and the standard deviation of at least 10 individually selected and analysed leaves. *F_v_*/*F_m_*: maximum PSII quantum yield; Φ_PSII_: effective PSII quantum yield. Stages 1-5 correspond (respectively) to the end of anthesis, the milk-development stage, the soft dough-development stage, the hard dough-development stage and the ripening stage.

**Figure 4:**
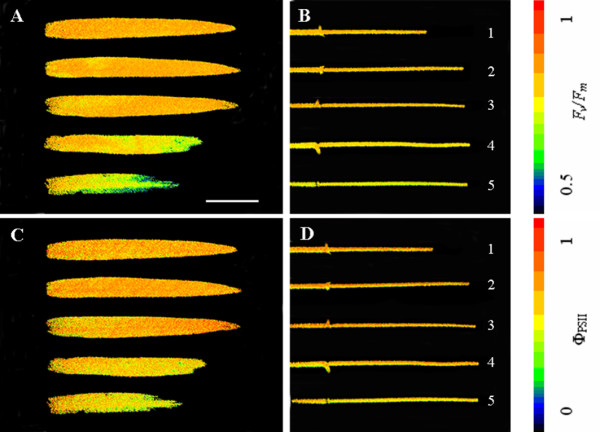
**Representative pseudocolour images showing *F_v_/F_m _*(A and B) and Φ_PSII _(C and D) of flag leaves (A and C) and exposed peduncles (B and D) at different stages**. Numbers 1-5 correspond (respectively) to the following developmental stages: the end of anthesis, the milk-development stage, the soft dough-development stage, the hard dough-development stage and the ripening stage. *F_v_*/*F_m_*: maximum PSII quantum yield; Φ_PSII_: effective PSII quantum yield. Bar = 5 cm.

The chlorophyll fluorescence parameters varied similarly in flag leaves and exposed peduncles during grain-filling. In the flag leaves, values of *F_v_/F_m _*and Φ_PSII _remained almost constant (0.807 ± 0.05 and 0.644 ± 0.04, respectively) at stages 1, 2 and 3 (Figure 3 and Figure 4). At stages 4 and 5, the values of *F_v_*/*F_m _*and Φ_PSII _in the flag leaves decreased sharply, and this was partly attributable to the occurrence of severe senescence initiated at the leaf tip (Figure 3 and Figure 4). In the exposed peduncles, both *F_v_/F_m _*and Φ_PSII _presented remarkably similar values at stages 1, 2 and 3. These values, which remained at 0.801 ± 0.1 and 0.632 ± 0.1, respectively, were slightly lower than those found in the flag leaves. In subsequent stages, the values steadily declined; however, the decline was slower than in the flag leaves. As a result, the values of *F_v_/F_m _*and Φ_PSII _in the exposed peduncles were slightly higher at stage 4, and significantly higher at stage 5, than those in the flag leaves. Thus, a relatively high level of PSII activity was maintained in the exposed peduncles during the late stages of grain-filling.

### PEPCase activity

**Figure 5:**
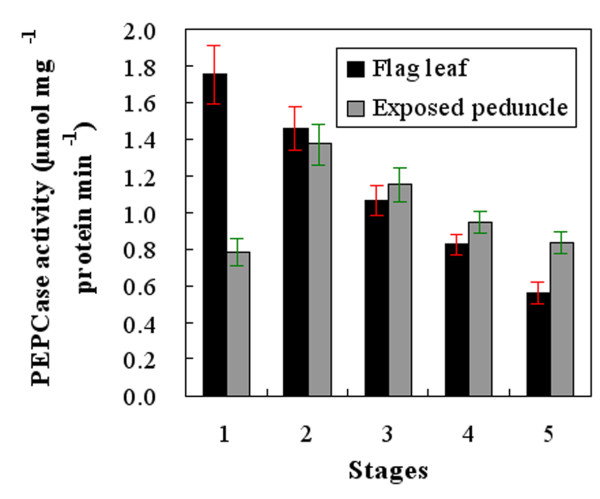
**Temporal changes in phospho*enol*pyruvate carboxylase (PEPCase) activity in flag leaves and exposed peduncles at different stages. Each PEPCase activity value represents the average and the standard deviation of five experiments**. Stages 1-5 correspond (respectively) to the end of anthesis, the milk-development stage, the soft dough-development stage, the hard dough-development stage and the ripening stage.

The maximum PEPCase activity in the flag leaves (1.75 μmol mg^-1 ^pro min^-1^) was observed at stage 1; this value then gradually decreased to 0.56 μmol mg^-1 ^pro min^-1 ^at stage 5 (Figure 5). In the exposed peduncles, the PEPCase activity was only 0.86 μmol mg^-1 ^pro min^-1 ^at stage 1, which was significantly lower than that in the flag leaves at this stage. The activity increased and reached its maximum of 1.37 μmol mg^-1 ^pro min^-1 ^at stage 2, close to that observed in the flag leaves. Towards stage 5, the PEPCase activity began to decline, but the decline was lower than in the flag leaves. As a result, the exposed peduncle PEPCase activity was slightly higher than that in the flag leaves at stages 3 (1.15 compared to 1.07) and 4 (0.95 compared to 0.82), and it was significantly higher at stage 5 (0.83 compared to 0.56; *P *< 0.05) (Figure 5).

### Photosynthetic contribution of the exposed peduncle to grain growth

No difference in grain mass was found between darkened and non-darkened plants from the commencement of grain-filling at stage1 and 2 (Table 2). A slight reduction in grain mass in the darkened wheat was observed at stage 3, and the reduction was found to be significant at stage 4 (*P *< 0.05) and at stage 5 (*P *< 0.01).

**Table 2:** Effect of darkening the exposed peduncle at the end of anthesis on grain dry mass (g ear^-1^) during grain-filling.

Treatment	Developmental stage				
	
	1^1^	2	3	4	5
Non-darkened (control)	0	0.12 ± 0.01a^2^	0.61 ± 0.04a	1.14 ± 0.05a*^3^	1.38 ± 0.06a**
Exposed peduncle darkened	0	0.12 ± 0.01a	0.59 ± 0.03a	1.07 ± 0.04b	1.26 ± 0.06b

^1^Stages 1-5 correspond to the end of anthesis, the milk-development stage, the soft dough-development stage, the hard dough-development and the ripening stage, respectively.

^2 ^Means followed by different letters within a column are significantly different by a Tukey test (*P *< 0.05).

^3 ^* and ** indicate significance at the 0.05 and 0.01 probability levels, respectively.

## Discussion

In this study, we observed that stomata occurred over the chlorenchyma strands at a higher density in exposed peduncles than in flag leaves. Stomata are involved in two of the most important plant processes, photosynthesis and transpiration [20]. A higher stomatal density would ensure a higher photosynthetic efficiency even at sub-optimal CO_2 _concentrations [20]. The higher stomatal density in exposed peduncles may therefore make an important contribution to the photosynthetic efficiency of the plant by increasing the surface area for gas exchange and improving the photosynthetic conversion of solar energy. In addition, the higher stomatal density in exposed peduncles might improve plant viability in hot environments (such as in China) at the late stages of grain-filling because of the cooling effect of stomatal transpiration on the peduncle and the canopy [21-23].

Recently, chloroplasts that are present in non-foliar organs have been found to be of have been found to be functionally important for photosynthesis and consequently also for grain yield in wheat [24]. High photosynthetic activity is characterised by well-developed chloroplasts with a high proportion of grana [25]. Because the integrity of cellular organelles and well-preserved membrane systems are essential for photosynthesis [13,14], a close relationship is expected to exist between chloroplast ultrastructure and the photosynthetic capacity during grain growth. In our study, we found that the chloroplasts in flag leaves had well-organised thylakoids and a well-organised structure at stages 1 and 2. At subsequent growth stages, the thylakoid membranes degraded and the starch granule population declined concomitantly with an increase in plastoglobuli, indicating that leaf senescence had commenced [19]. In exposed peduncles, the chlorenchyma cells contained a large number of chloroplasts, each developing numerous grana with a high proportion of granal stacks at stages 1, 2 and 3. Given that the chloroplasts in the exposed peduncles remained more structurally conserved at stages 4 and 5 (as compared with the ruptured ones in the flag leaves), it is reasonable to propose that exposed peduncles remain functionally active during grain-filling. The exposed peduncle makes additional contributions to assimilate production, especially when the flag leaf senesces quickly and its photosynthetic activity declines. As one of the photosynthetically active organs, the exposed peduncle also has the advantage of being located nearer to the grains than the flag leaf. Thus, the photosynthetic assimilates are directly transferred to the grain along a short path.

*F_v_*/*F_m _*and Φ_PSII _are important indicators of photosynthetic capacity. PSII efficiency has also been shown to be an effective indicator of leaf senescence in plants [26]. In this study, the results for *F_v_*/*F_m _*and Φ_PSII _demonstrated that the rates remained relatively constant in both organs; no significant differences were observed between these two organs at stages 1, 2 and 3, indicating that the exposed part of the peduncle had efficient light utilisation by PSII. However, the rates in the exposed peduncles declined relatively slowly thereafter, in contrast to the dramatic decreases in the flag leaves. As a result, the rates were higher in the exposed peduncles than in the flag leaves at stages 4 and 5. It has been demonstrated that natural and/or stress-induced senescence causes a loss of photosynthetic capacity [26]. The slower decline in PSII efficiency thus suggests that the exposed peduncle senesces more slowly than the flag leaf and can continue to function longer during grain-filling, and this may be important for maintaining grain growth at late stages. Because PSII is mostly located in the granal thylakoids of the chloroplast [14], the reduction in PSII efficiency might be correlated with reduced thylakoid integrity. This hypothesis has been confirmed by TEM observation of the ultrastructure of chloroplasts as described above.

Of particular interest is the observation that the values of *F_v_*/*F_m _*and Φ_PSII _remained high in both exposed peduncle and flag leaf even at stage 3. It is tempting to suggest that the higher than expected PSII efficiency is associated with greater source strength at the mid-grain-filling stage.

During grain-filling, PEPCase is more important for maintaining carbon assimilation activity in the non-foliar organs than ribulose bisphosphate carboxylase (RuBP carboxylase, EC 4.1.1.39) [16,22]. The PEPCase activity in non-foliar organs has therefore been considered an important biochemical characteristic of wheat plants with high photosynthetic efficiency [22]. In our study, we found that the variation in PEPCase activity over time differed between the flag leaf and the exposed peduncle. At stages 1 and 2, the activity of this enzyme was lower in the exposed peduncle, whereas the activity was higher at late stages, especially stages 4 and 5. Because PEPCase supplies substrates for carbohydrate synthesis and its activity in the exposed peduncle is high during late grain-filling, the exposed peduncle may play an important role in assimilate production, especially at the late stage of grain growth. Given that PEPCase activity is closely associated with the variation in photosynthetic heat tolerance among organs of the same plant and that high temperature stress occurs frequently during late grain-filling in northern China [21,22], it is reasonable to postulate that the higher PEPCase activity in the exposed peduncle may lend it better photosynthetic thermotolerance than the flag leaf. This would confer an ecological advantage to wheat plants under warmer conditions.

Using organ-darkening treatments, the relative contributions of the green organs to yield grain can be estimated [8,18]. In the present study, no difference in grain mass was found between darkened and non-darkened plants at stages 1 and 2. The photosynthates produced prior to the treatment were sufficient to meet the relatively lower requirement of the weaker sink strength in the early stages of grain development. At late developmental stages, we observed significant reductions in grain growth in dark-treated wheat (Table 2). The difference in grain mass between the exposed peduncle darkening treatment and the control could be due to the reduced availability of photoassimilates from the exposed peduncle. We therefore hypothesise that assimilates from the exposed green part of the peduncle make an important contribution to grain growth.

## Conclusion

We found that the exposed peduncle possesses anatomical, ultrastructural and physiological advantages over the flag leaf for performing photosynthesis. These advantages are especially obvious in the later stages of grain-filling. The higher stomatal density and the higher activity of heat-tolerant PEPCase revealed that the exposed peduncle has a superior ability to adapt to the ecological environment at the later stages of grain-filling. Based on the data presented here, we conclude that the exposed peduncle has a strong photosynthetic capacity and provides assimilates for the development of grain mass during grain-filling.

## Methods

### Plant materials

Winter wheat (*Triticum aestivum *L.) Jimai 22 was sown on October 8, 2008 at an experimental station (36°42' N, 117°4' E; altitude 48 m) at the Shandong Academy of Agricultural Sciences, China. Based on grain development, five growth stages were designated for measurement: stage 1 (the end of anthesis) occurred on 30 April; stage 2 (milk-development stage) occurred on 8 May; stage 3 (the soft dough-development stage) occurred on 16 May; stage 4 (the hard dough-development stage) occurred on 24 May; and stage 5 (the ripening stage) occurred on 2 July. Samples of the flag leaf blades and the exposed peduncles were collected at each stage. The peduncle length and diameter were measured at stages 1 and 2.

### Scanning electron microscopy

The mid-portions of the flag leaf blades and the exposed peduncles were measured to ensure the uniformity of the sample material. Samples were cut into 3-mm long sections and were fixed in 2.5% glutaraldehyde solution in 100 mM sodium phosphate buffer (pH 7.2) for more than 24 h, dehydrated in a graded ascending series of ethanol, and critical-dried in a CO_2 _atmosphere. The samples were then mounted on aluminium stubs using double-sided adhesive tape. After mounting, the samples were sputtered with gold (~20 nm). The stomatal morphology and distribution on the surface of the specimens were observed using a scanning electron microscope (SEM; XL30, Philips, Eindhoven, The Netherlands). The stomatal density was measured from the SEM images of the samples. Each value represents the mean of at least ten replicates.

### Light and transmission electron microscopy

Samples of four flag leaf blades and four exposed peduncles were collected randomly from four plants. The collected samples were immediately fixed in 2.5% glutaraldehyde solution in 100 mM sodium phosphate buffer (pH 7.2) and stored overnight at room temperature. After washing with 100 mM sodium phosphate, the samples were post-fixed with 1% (w/v) osmium tetroxide in phosphate buffer at 4°C for 2 h. The samples were then dehydrated in an ethanol series, transferred into propylene oxide and finally embedded in Epon812 (Shell Chemical, Houston, TX, USA). Four 2-μm thick sections were cut with an LKB-V microtome from each sample. The sections were mounted on microscope slides and stained with toluidine blue O. The chloroplast structure and the stomata were observed and photographed using an optical microscope (Zeiss Axioskop 40, Leica, Germany) equipped with a digital camera. Ultrathin sections were cut with an LKB-V microtome and then mounted on a formvar-coated brass grid. The sections were stained with 2% uranyl acetate (w/v) in 70% methanol (v/v) and 0.5% lead citrate. The ultrastructure and the shape of the chloroplasts were observed with a JEM-1230 transmission electron microscope (TEM; JEOL Ltd., Tokyo, Japan) at 80 kV.

### Chlorophyll fluorescence assay and imaging

Chlorophyll fluorescence analysis was performed at different stages to determine the maximum PSII quantum yield (*F_v_*/*F_m_*) and the PSII effective quantum yield (Φ_PSII_) in the flag leaves and exposed peduncles.

The chlorophyll fluorescence induction kinetics (Kautsky effect) in pre-darkened leaves and peduncles (20 min) were measured at the red chlorophyll fluorescence band (near 690 nm) using a kinetic imaging fluorometer (FluorCam, Photon System Instruments Ltd., Brno, Czech Republic) as described by Nedbal et al. (2000) [27]. The duration of the *F_0 _*measurement was 5.04 s. After measuring the minimum fluorescence in the dark-adapted state (*F_0_*), the samples were illuminated with a saturating pulse (1500 μE m^2 ^s^-1^) to determine the maximal fluorescence in the dark-adapted state (*F_m_*). For quenching analysis, samples were illuminated after 10 s of darkness with an orange actinic light (130 μE m^2 ^s^-1^) using saturating pulses of 60 s. The fluorescence values that were recorded at every saturating pulse were identified as *F_m_*', whereas the fluorescence values recorded immediately prior to each pulse were called *F_t_*. The chlorophyll fluorescence emission transients were captured by a CCD camera in a series of images with a resolution of 512 × 512 pixels to reveal the heterogeneity of the leaf surface. Numerical analyses of the classical physiological parameters were performed on the maximum PSII quantum yield *F_v_*/*F_m _*= (*F_m _*- *F_0_*)/*F_m _*and on the effective PSII quantum yield Φ_PSII _= (*F_m_*' - *F_t_*)/*F_m_*' using more than ten replicates per organ [2].

### PEPCase extraction and activity assays

The enzyme extraction was carried out according to Vu *et al*. (1985) with slight modifications. Samples of the flag leaf blades or the exposed peduncles (0.2 g) were collected and immediately ground into a fine power with a mortar and pestle containing liquid nitrogen. The powder was transferred into 10 ml of extraction medium that consisted of 50 mM Tris-HCl (pH 7.8), 5 mM DTT, 100 mM MgC1_2_, 1 mM EDTA, and 2% (w/v) soluble polyvinylpyrrolidone (PVP-40). Following centrifugation at 15,000 *g *for 10 min at 4°C, the supernatant was used for enzyme assays.

The PEPCase activity was measured spectrophotometrically at 340 nm using a UV/Vis spectrophotometer (Beckman DU 800, USA) and by coupling the PEP reaction to the oxidation of NADH with malate dehydrogenase (MDH) according to Blanke and Ebert (1992) [15]. The enzyme extract was added into a solution to a final volume of 1 cm^3 ^and contained 50 mM Tris-HCl (pH 8.2), 10 mM MgCl_2_, 0.25 mM EDTA, 5.0 mM NaHCO_3_, 2.0 mM DTT, 4 units MDH, 0.1 mM NADH, and 2.0 mM PEP. The reaction was started by the addition of tissue extract and OD340 of samples was measured against a blank consisting of substrate and buffer but with no plant extract. The water soluble protein was determined using the Bradford (1976) assay [28]. Data were averaged from five replicates.

### Photosynthetic contribution of the exposed peduncle to grain growth

To investigate the photosynthetic contribution of the exposed peduncle to grain growth, 300 plants with exposed peduncles of about the same length were selected at stage 1. Limitation of the photosynthesis of (and thus the assimilate supply from) the exposed peduncles was achieved in 150 selected plants by wrapping the exposed peduncles in aluminium foil with 1-mm-diameter holes accounting for about 0.3% of the covered area to prevent the accumulation of ethylene and water vapour [8]. The samples were rewrapped every two days using longer strips of aluminium foil until the exposed peduncles were fully elongated (eight days after anthesis). Spikes were harvested from the control (no darkening covers) and the darkened plants at eight-day intervals after the darkening treatment and were then oven dried at 60°C for 48 h and manually threshed. Grains were weighed to calculate the yield per plant. Data were averaged from five replicates, each with six spikes.

### Statistical analysis

All of the data were subjected to an analysis of variance (ANOVA) using DPS statistical software (v.7.55, Refine Information Tech. Co., Ltd., Hangzhou, Zhejiang, China). The data are presented as the mean ± standard deviation. The significance of differences between mean values was determined with Tukey's test. Differences at *P *< 0.05 were considered significant.

## Authors' contributions

LK was responsible for the experiments, the semithin and ultrathin section preparations, the data analysis and drafting the manuscript. FW conceived and designed the study and helped draft the manuscript. JS carried out the PSII quantum yield assays and helped draft the manuscript. BF established the PEPCase activity assay. SL and BZ performed the estimation of the photosynthetic contribution of the exposed peduncle on grain growth and made the morphological observations. All authors read and approved the final manuscript.

## Acknowledgements

This research was supported by the CGIAR's Challenge Program on Water and Food (CPWFYRB200501) and the High-Technology Independent Innovation Foundation of Shandong Academy of Agricultural Sciences (2006YBS025, 2007YCX024).
